# Prevalence and Predictive Factors of Cervical Cancer Screening in Saudi Arabia: A Nationwide Study

**DOI:** 10.7759/cureus.49331

**Published:** 2023-11-24

**Authors:** Fatimah H Alkhamis, Zainab Alabbas S Alabbas, Jwaher E Al Mulhim, Fadk F Alabdulmohsin, Mariyyah H Alshaqaqiq, Eithar A Alali

**Affiliations:** 1 Obstetrics and Gynaecology, King Faisal University, Alhofuf, SAU

**Keywords:** human papillomavirus, epidemiology, saudi arabia, hpv, screening, cervical cancer

## Abstract

Background: Cervical cancer, despite being preventable, is one of the most prevalent cancers among females globally and in Saudi Arabia. The literature demonstrated that, unlike global trends, cervical cancer incidence in Saudi Arabia is increasing. In addition to that, a high proportion of it is discovered in advanced stages. This state of late discovery was attributed to the absence of efficient preventive and screening programs. Observing the scale of the preventable morbidities and mortalities that can be caused by cervical cancer and the efforts and costs that are positioned to fight cervical cancer across the globe and the current ambiguity in the cervical cancer screening prevalence in Saudi Arabia brings the realization of the importance of conducting a study that properly explores the status of cervical cancer screening in Saudi Arabia.

Aim: This study aims to measure the prevalence and the predictive factors for cervical cancer screening among adult women who were previously sexually active in Saudi Arabia, as well as explore the participants' knowledge and attitude toward cervical cancer and human papillomavirus (HPV) vaccines.

Methods: This study was a nationwide cross-sectional study conducted in Saudi Arabia between September 2023 and November 2023 on adult Saudi females between the ages of 21 and 65 years who were previously sexually active and did not undergo a hysterectomy. Data were collected through a convenience sampling technique where a self-administered survey was established and disseminated to the targeted population all over the country with the assistance of data collectors. Cervical cancer screening prevalence and screening predictive factors were measured. Factors associated with cervical cancer screening and knowledge of cervical cancer were tested using a chi-square test, an independent t-test, and an ANOVA test. Multivariate logistic regression was also used to determine predictors of cervical cancer screening.

Results: The study included 2,337 participants. The prevalence of cervical cancer screening among Saudi females was observed to be 22.1%. The most commonly reported reason for not undergoing cervical cancer screening was that it was never recommended by a physician, as reported by 42.4%. Only 7.6% reported taking the HPV vaccine. The majority of the participants (84.1%) had a low knowledge level about cervical cancer. The multivariate logistic regression model revealed that the following factors were observed to be significantly predictive of undergoing cervical cancer screening: being 46-59 years of age (74% increase rate), having an income greater than 20,000 Saudi Riyals (SRs) (158% increase rate), having a history of gynecological problems (152% increase rate), knowing someone who underwent cervical cancer screening (393% increase rate), and receiving a recommendation from a healthcare practitioner to undergo cervical cancer screening (1300% increase rate).

Conclusion: There are clearly low rates of cervical cancer screening and even lower rates of uptake for the HPV vaccine, which are the prevention measures for cervical cancer. National initiatives and programs that promote HPV vaccine uptake and regular cervical cancer screening are highly recommended to minimize the morbidity and mortality of cervical cancer.

## Introduction

Cancer remains a serious public health concern, as it is the second-leading cause of mortality worldwide [1]. Cervical cancer, despite being preventable, is the fourth most prevalent cancer among females globally, and in Saudi Arabia, cervical cancer is the ninth most common cancer among adult females [1-2]. The global incidence of cancer in 2020 was estimated to be around 19 million, and the global incidence of cervical cancer was about 600,000, constituting 6.9% of all cancers affecting females [3]. In Saudi Arabia, the incidence of cervical cancer in 2020 was 358 (the crude incidence per 100,000 women was 2.4 and the mortality incidence ratio was 0.5) [4-5]. By 2030, the yearly incidence of cervical cancer is expected to jump to 700,000, and the number of deaths is predicted to increase to 400,000 if no further intervention is done [6]. Although the global trends of cervical cancer have shown a consistent decline, in Saudi Arabia, cervical cancer incidence has increased by 453.6% since 1990, with a yearly mortality of 179 [7-8].

Cervical cancer is characterized by an abnormal growth of cells on the lining of the cervix, with two main types. Squamous cell carcinoma (SCC) accounts for 70% of all cervical cancer cases, and adenocarcinoma accounts for 25% of all cervical cancer cases [9]. The primary cause of cervical pre-cancer stage and cancerous transformation is chronic infection with an oncogenic type of human papillomavirus (HPV) [10]. Although HPV has more than 100 types, HPV 16 and 18 are the variants that lead to cervical cancer development [10]. Typically, cervical cancer occurs after a longstanding period of the pre-malignant stage, termed cervical intraepithelial neoplasia (CIN), which can last up to 10 years before progressing to invasive cancer [11]. The early presenting symptoms of cervical cancer include irregular bleeding, post-coital spotting, vaginal discharge, and pre-or post-menopausal bleeding. In advanced stages, urinary urgency, frequency, lower abdominal pain, back pain, and weight loss can occur [10]. Although HPV is the primary cause of cervical cancer, other risk factors like starting sexual activity at an age younger than 21 years, smoking, multiple sexual partners, a history of sexually transmitted disease, three or more full-term pregnancies (high parity), and five years and more of hormonal contraceptives have been identified.

This pre-malignant time window, which can last up to 10 years, provides sufficient time for screening and early intervention that can significantly improve outcomes [12]. Cervical cancer screening can be done using a pap smear, as it can reveal cytological abnormalities in the cervical transformation zone. This method of screening helped in achieving a 70% decrease in the incidence and mortality of cervical cancer in developed countries after three years of implanting the screening program [13-15]. A recommendation by the United States Preventive Services Task Force was made to screen women aged between 21 and 65 using pap smears every three years. For those wishing to increase the length of the screening interval to every five years, it was recommended to add the HPV test, which is applied only to those aged 30 to 65 years [16]. As another countermeasure for cervical cancer, the Advisory Committee on Immunization Practices in the United States made a recommendation to give girls between the ages of 11 and 26 the HPV vaccine [17].

Due to the preventable nature of the disease and the mortality and morbidity caused by it, the World Health Organization (WHO) scaled up its efforts against cervical cancer and launched the Global Strategy to Accelerate the Elimination of Cervical Cancer, which includes three crucial elements: vaccination, screening, and treatment. This strategy is predicted to decrease the incidence by 40% and prevent five million deaths by 2050 if its implementation succeeds [1, 6]. In England, a huge reduction in cervical cancer rates was recorded after the implementation of the HPV vaccination program [18]. In addition to that, a 51%-92% decline in cervical cancer mortality was recorded in different parts of Europe after the application of the cervical cancer screening program [19]. The effectiveness of these programs depends heavily on the level of the population's knowledge and acceptance. A systematic review previously reported that educational intervention improved the rate of cervical cancer remarkably [2].

In Saudi Arabia, more than 40% of cervical cancer cases are discovered at advanced stages (III and IV), compared to 25% of cases in the province of British Columbia in Canada. This high rate of late discovery was attributed to the absence of efficient preventive and screening programs [20-21]. A study in 2022 in Jeddah showed a cervical screening rate of 33.4% [22], and a study in Riyadh in 2019 showed a cervical cancer screening rate of 26% [23]. Another study conducted in 2009 in Jeddah found that the rate of cervical cancer screening using a pap smear was only 16.8% [24]. Another study conducted in 2019 that surveyed four Gulf countries showed a cervical cancer screening rate of 7.6% in Saudi Arabia, 10.6% in Oman, 17.7% in Kuwait, and 28% in the United Arab Emirates [25]. These works reflect an overall low rate of cervical cancer screening in Saudi Arabia; however, they lack consistency of results and lack appropriate nationwide coverage of the targeted population to precisely measure the true rate of cervical cancer screening.

Previous studies demonstrate several factors that can affect cervical cancer uptake and thus hinder the achievement of the targeted goal of the WHO strategy. Age, marital status, level of income, level of education, place of residency, employment status, and failure of proper delivery of information from the health care provider to the patients about the benefit of cervical cancer screening were all found to be associated with cervical cancer screening uptake [26-28]. It was observed that higher income levels, higher levels of education, and being employed were all associated with higher uptake for screening [27-29]. Undergoing a gynecological examination in the past and the recommendation of a family doctor were also perceived as factors associated with undertaking cervical cancer screening [22].

Observing the scale of the preventable morbidities and mortalities that can be caused by cervical cancer and the efforts and costs that are positioned to fight cervical cancer across the globe and the current ambiguity in the cervical cancer screening prevalence in Saudi Arabia brings the realization of the importance of conducting a study that properly explores the status of cervical cancer screening in Saudi Arabia. This study aims to measure the prevalence and predictive factors for cervical cancer screening among adult women in Saudi Arabia who were previously sexually active, as well as explore the participants' knowledge and attitude about cervical cancer and HPV vaccines. Enriching the literature with reliable information about the prevalence and predictive factors of cervical cancer screening in Saudi Arabia, as well as knowledge and attitude toward cervical cancer and the HPV vaccine, by conducting a nationwide study will help bring insight into the current status, which can guide the setting forth of a comprehensive national comprehensive plan to eliminate cervical cancer in Saudi Arabia.

## Materials and methods

Study design and settings

This study had a cross-sectional design. It targeted adult Saudi females between the ages of 21 and 65 who had been previously sexually active to measure the prevalence and predictive factors of cervical cancer screening, as well as their knowledge and attitude toward cervical cancer and the HPV vaccine. The study was conducted between September 2023 and November 2023.

Study subjects, inclusion, and exclusion criteria

The study subjects were adult Saudi females ranging in age from 21 to 65 who had been sexually active before and were living in Saudi Arabia, who consented to participate in the study, and who met the inclusion and exclusion criteria.

The inclusion criteria consisted of being an adult Saudi female aged between 21 and 65 years, being previously sexually active, living in Saudi Arabia, not having undergone a hysterectomy, and consenting to participate in the study.

The exclusion criteria consisted of being younger than 21 years old or older than 65 years old, never having been sexually active, being non-Saudi or living outside of Saudi Arabia, having undergone a hysterectomy before, and not consenting to participate in the study.

Study tool and its validation

A questionnaire was developed by the investigators and presented to multiple consultants in the obstetrics and gynecology departments to improve. The survey was approved after the modifications were done by the experts in the field. The questionnaire was initially constructed in English and then translated by a certified linguist to Arabic for it to be understandable by the targeted population. Two other language experts then examined the Arabic version of the survey, and the translation was approved after grammatical and linguistic modifications. After that, a pilot study was performed on a small number of people (18) to ensure a uniform comprehension of the questionnaire content.

Sampling and sample size

Convenience random sampling was employed as a sampling technique, where the survey was scattered and spread within all regions of Saudi Arabia and the targeted population was invited to participate through a link. The sample size was calculated using the following formula: n = z2pq\d 2. The confidence level was set at 99.9%, the estimated proportion was set at 50%, and the level of precision was set at 3%. The target population (females aged 21 to 65 years old), in accordance with the latest published numbers by the Saudi National Authority of Statistics, was estimated to be nearly six million [30]. The minimum calculated sample size was 1,691; despite that, more participants were enrolled to secure high sufficiency and accuracy of results.

Data collection

An online questionnaire was established through Google Forms (Google Inc., Mountain View, CA) for collecting data. The online questionnaire was disseminated to the targeted population within each of the five main areas of Saudi Arabia, respectively (central, eastern, northern, western, and southern areas). The target population was invited to participate voluntarily after consenting. In order to reach the target population in each of the five areas and for the sake of enrolling as many participants as possible, five data collectors were recruited from every area. The data collection process occurred through a self-administered questionnaire where the participants initially gave consent to participate in the study before starting to fill out the survey. The questionnaire consisted of four sections. The first section inquired about sociodemographic data and medical history. The second section explored participants’ previous experience with cervical cancer screening and the HPV vaccine. The third section investigated the participants’ knowledge of cervical cancer screening and the HPV vaccine. The fourth section evaluated the participants’ attitudes about cervical cancer screening and the HPV vaccine.

Statistical analysis

Data analysis was performed using IBM SPSS software, version 23 (IBM Corp., Armonk, NY). Frequency and percentages were used to display categorical variables. Minimum, maximum, mean, and standard deviation were utilized to present numerical variables. A chi-square test was used to test for factors associated with undergoing cervical cancer screening. Multivariate logistic regression was used to determine the predictors of undergoing cervical cancer screening. The logistic regression model included the following variables: age, education, marital status (single participants were not included in the model due to the possibility of cultural barriers for single participants to undergo this examination), working status, income, place of residency, previous diagnosis with sexually transmitted disease, having a chronic disease, smoking, history of gynecological problems, knowing someone who underwent cervical cancer screening, knowing someone who had cervical cancer, receiving a recommendation from a healthcare practitioner to undergo cervical cancer screening, and knowledge levels of cervical cancer. The goodness-of-fit for the model was tested using the Omnibus test and the Hosmer and Lemeshow test. An independent t-test and an ANOVA test were used to test factors associated with the knowledge score. The level of significance was set at 0.05. For the sake of knowledge assessment, each question related to knowledge was assigned a score, where participants choosing a correct answer granted them a score, and participants not choosing correctly or choosing a wrong answer granted them a score of 0. The lowest possible score was 0, and the highest was 13. Furthermore, participants' knowledge was stratified into levels, where those who got less than 50% of the total score (six or less) were considered to have a low knowledge level, those scoring 50%-75% of the total score (seven to nine) were considered to have a moderate knowledge level, and those scoring more than 75% of the total score (10 and more) were considered to have a high knowledge level.

Confidentiality and ethical considerations

The participants' confidentiality and the privacy of their data were given high priority. No personal data or contact information were requested from the participants. Ethical approval was obtained from the ethical committee in King Faisal University's Deanship of Research at Alhofuf, Saudi Arabia. Written consent was obtained from all participants before being part of the study.

## Results

A total of 2,337 participants were included in the study. Table 1 shows the sociodemographic profile of the participants.

**Table 1:** Sociodemographic profile of the participants (n = 2337) SR: Saudi Riyal

Sociodemographic characteristics	n	%
Age		
21-30 years	746	31.90
31-45 years	815	34.90
46-59 years	627	26.80
60-65 years	149	6.40
Education		
Intermediate school or less	73	3.10
High school	401	17.20
Bachelor’s degree or diploma	1656	70.90
Higher education (Masters/PhD)	207	8.90
Marital status		
Single	401	17.20
Married	1677	71.80
Divorced	172	7.40
Widowed	87	3.70
Working status		
Working	994	42.50
Not working	1066	45.60
Retired	277	11.90
Income		
Less than 5,000 SR	351	15.00
5,000-10,000 SR	706	30.20
10,001-15,000 SR	545	23.30
15001-20000 SR	360	15.40
Above 20,000 SR	375	16.00
Place of residence in Saudi Arabia		
Northern region	190	8.10
Eastern region	571	24.40
Western region	432	18.50
Southern region	284	12.20
Central region	860	36.80

Of the sample, 746 (31.9%) of the participants were between 21 and 30 years, 815 (34.9%) were between 31 and 45 years, 627 (26.8%) were between 46 and 59 years, and 149 (6.4%) were between 60 and 65 years. As for education, 73 (3.1%) had an intermediate school education or less, 401 (17.2%) had a high school education, 1,656 (70.9%) had a bachelor’s degree or diploma, and 207 (8.9%) had a higher education (Masters or PhD). In regard to marital status, 401 (17.2%) were single, 1,677 (71.8%) were married, 172 (7.4%) were divorced, and 87 (3.7%) were widowed. Regarding employment status, 944 (42.5%) were working, 1,066 (45.6%) were not working, and 277 (11.9%) were retired. As for income, 351 (15%) had an income less than 5,000 Saudi Riyal (SR), 706 (30.2%) had an income between 5,000 and 10,000 SR, 545 (23.3%) had an income between 10,001 and 15,000 SR, 360 (15.4%) had an income between 15,001 and 20,000 SR, and 375 (16%) had an income above 20,000 SR. Regarding their place of residence, 190 (8.1%) were from the northern region, 571 (24.4%) were from the eastern region, 432 (18.5%) were from the western region, 284 (12.2%) were from the southern region, and 860 (36.8%) were from the central region.

Table 2 displays the medical and gynecological history of the study group.

**Table 2:** Medical and gynecological history of the study group (n = 2337)

Question	n	%
Medical history		
Do you have any chronic disease (diabetes, hypertension, asthma, or any other chronic disease)?		
Yes	549	23.5
No	1788	76.5
Do you smoke (cigarettes, vape, or hookah)?		
Yes	268	11.5
No	2069	88.5
Gynecological history		
Have you had any gynecological problems before (abnormal vaginal bleeding, vaginal discharge, or other problems)?		
Yes	1245	53.3
No	1092	46.7
Have you ever been diagnosed with any sexually transmitted disease?		
Yes	114	4.88
No	2223	95.12

As for medical history, 549 (23.5%) reported having a chronic disease (diabetes, hypertension, asthma, or any other chronic disease), while 1788 (76.5%) did not. Among the study group, 268 (11.5%) reported being smokers, while 2,069 (88.5%) reported they were not. Regarding their gynecological history, 1,245 (53.3%) reported having had a history of gynecological problems (abnormal vaginal bleeding, vaginal discharge, or other problems), while 1,092 (46.7%) did not. Among the participants, 114 (4.88%) reported being previously diagnosed with sexually transmitted disease, while 2,223 (95.12%) never were.

**Figure 1:**
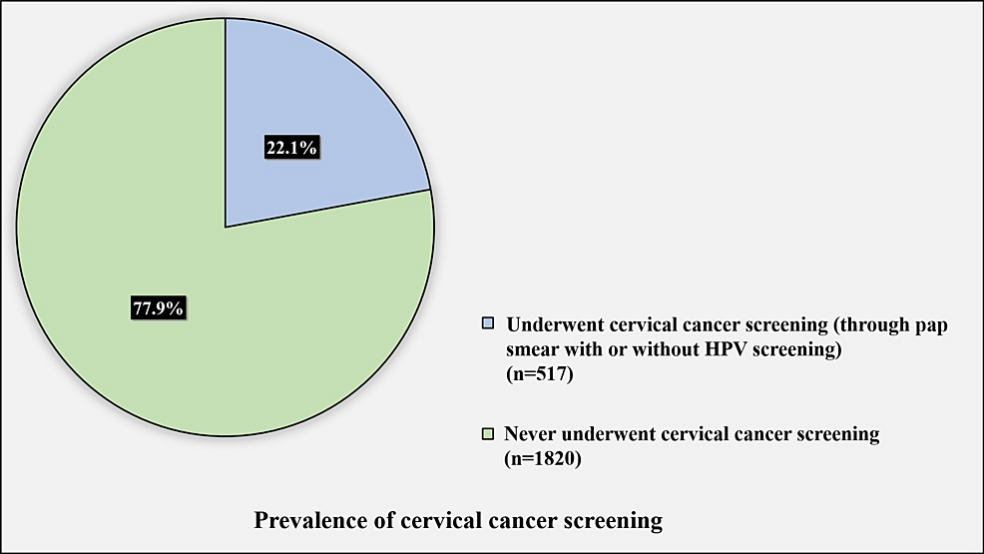
Prevalence of cervical cancer screening HPV: human papillomavirus

Figure 1 presents the prevalence of cervical cancer screening.

Only 517 (22.1%) of the participants underwent cervical cancer screening (through pap smear with or without HPV screening), while 1,820 (77.9%) never underwent cervical cancer screening.

**Figure 2:**
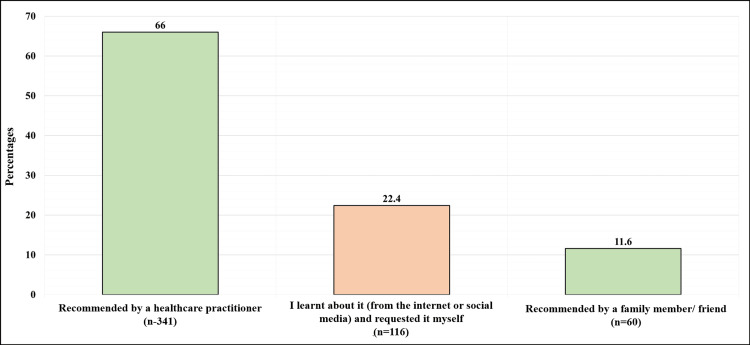
The reasons why participants underwent cervical cancer screening (n = 517)

Figure 2 demonstrates the participants' reasons for undergoing cervical cancer screening (n = 517). Among those who underwent the procedure, 341 (66%) reported it was because of a healthcare practitioner's recommendation, 116 (22.4%) reported it was because they learned about it and requested it for themselves, and 60 (11.6%) reported it was because of a family or friend's recommendation.

**Figure 3:**
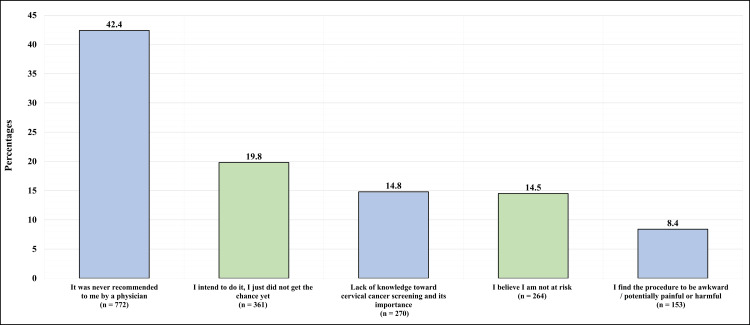
The reasons given by the participants for not undergoing cervical cancer screening (n=1820)

Figure 3 illustrates the reasons why participants (n=1820) did not undergo the procedure of cervical cancer screening.

Of the group, 772 (42.4%) reported that it was never recommended to them by a physician, 361 (19.8%) reported that they intended to do it but did not get the chance to do it, 270 (14.8%) reported a lack of knowledge about cervical cancer screening and its importance, 264 (14.5%) reported that they believe they are not at risk, and 153 (8.4%) reported that they find the procedure to be awkward, potentially painful, or harmful.

**Figure 4:**
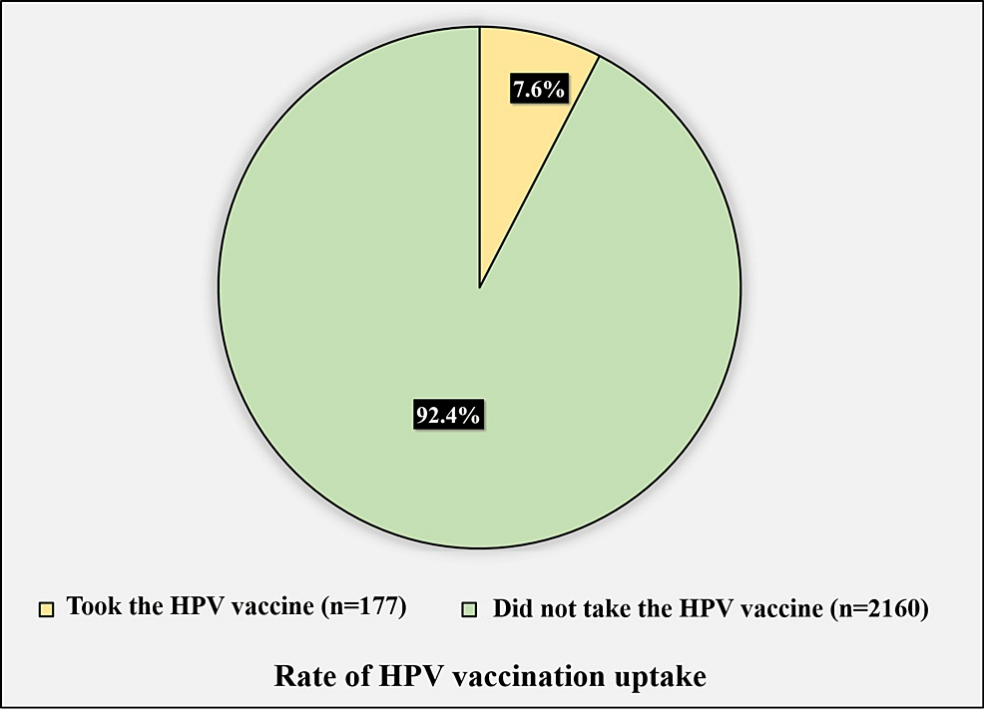
Rate of HPV vaccination uptake among the participants HPV: human papillomavirus

Figure 4 shows the rate of HPV vaccination uptake.

Only 177 (7.6%) participants reported taking the HPV vaccine, while 2,160 (92.4%) reported they did not.

Table 3 displays the participants' attitudes and previous experience with cervical cancer screening and HPV vaccination.

**Table 3:** Participants’ attitudes and previous experiences with cervical cancer screening and HPV vaccination (n = 2337) HPV: human papillomavirus

Question	n	%
Previous experience with cervical cancer screening and HPV vaccination		
Do you know someone who underwent cervical cancer screening?		
Yes	655	28
No	1682	72
Do you know someone who had cervical cancer?		
Yes	405	17.3
No	1932	82.7
Have you ever received a recommendation from a healthcare practitioner to undergo cervical cancer screening?		
Yes	586	25.1
No	1751	74.9
Have you ever received a recommendation from a healthcare practitioner to take the HPV vaccine?		
Yes	292	12.5
No	2045	87.5
Attitude towards cervical cancer screening and HPV vaccination		
If a doctor recommends it to you, would you undergo cervical cancer screening?		
Yes	1986	85
No	351	15
Knowing that the HPV vaccine can prevent cervical cancer, if you were eligible to take it and it was offered to you, would you take it?		
Yes	1881	80.5
No	456	19.5
Knowing that the HPV vaccine can prevent cervical cancer, if it was offered to your children would you let them take it?		
Yes	1853	79.3
No	484	20.7

As for the previous experience with cervical cancer screening and HPV vaccination, only 655 (28%) reported knowing someone who underwent cervical cancer screening, and 405 (17.3%) reported knowing someone who had cervical cancer. Only 586 (25.1%) reported previously receiving a recommendation from a healthcare practitioner to undergo cervical cancer screening, and only 292 (12.5%) reported receiving a recommendation from a healthcare practitioner to take the HPV vaccine. When participants were asked if they would undergo cervical cancer screening if a doctor recommended it, 1,986 (85%) answered positively, and 351 (15%) answered negatively. Of the group, 1,881 (80.5%) of the participants reported that knowing the HPV vaccine can prevent cervical cancer, they would take it if it was recommended for them and they were eligible to take it, while 456 (19.5%) reported they would not take it; 1,853 (79.3%) of the participants reported that knowing the HPV vaccine can prevent cervical cancer, they would give it to their children if it was recommended for them and they were eligible to take it, while 484 (20.7%) reported they would not take it.

Table 4 presents participants' knowledge assessments about cervical cancer.

**Table 4:** Participants’ knowledge assessment of cervical cancer (n = 2337) HPV: human papillomavirus

Question	n	%
1. To a high degree, cervical cancer is a preventable disease.		
True (correct answer)	1200	51.3
False	75	3.2
I don’t know	1062	45.4
2. The main cause of cervical cancer is HPV.		
True (correct answer)	824	35.3
False	92	3.9
I don’t know	1421	60.8
3. Which of the following is a sign or symptom of cervical cancer?		
Vaginal bleeding between periods (correct answer)	685	29.31
Abnormal menstrual bleeding (longer / heavier) (correct answer)	414	17.72
Pain during intercourse (correct answer)	447	19.13
Bleeding after intercourse (correct answer)	407	17.42
Pelvic pain (correct answer)	392	16.77
I don't know	1350	57.77
4. Which of the following is a risk factor for cervical cancer?		
Multiple sexual partners (correct answer)	814	34.83
Early start of sex life (correct answer)	216	9.24
High number of full-term pregnancies (correct answer)	121	5.18
Smoking (correct answer)	386	16.52
History of HIV or other sexually transmitted disease (correct answer)	727	31.11
Prolonged use of oral contraceptive pills (correct answer)	360	15.40
I don't know	1138	48.69
Knowledge score (lowest possible score = 0, highest possible score = 13)		
Minimum	0	
Maximum	13	
Mean	2.99	
Standard deviation	3.06	
Knowledge levels	n	%
Low knowledge level (a score of 6 or less) (less than 50% of the total score)	1965	84.10
Moderate knowledge level (a score between 7-9) (a score between 50%-75% of the total score)	269	11.50
High knowledge level (a score of 10 or more) (more than 75% of the total score)	103	4.40

Only 1,200 (51.3%) of the participants knew that cervical cancer, to a high degree, is a highly preventable disease, and only 824 (35.3%) of the participants knew that the main cause of cervical cancer is HPV. As for the knowledge score, the minimum score was 0, the maximum was 13, and the mean was 2.99+3.06. As for the knowledge level, 1,935 (84.1%) had a low knowledge level (a score of six or less) (less than 50% of the total score), 269 (11.5%) had a moderate knowledge level (a score between seven and nine) (a score between 50% and 75% of the total score), and 103 (4.4%) had a high knowledge level (a score of 10 or more) (more than 75% of the total score).

Table 5 demonstrates the factors associated with undergoing cervical cancer screening.

**Table 5:** Factors associated with undergoing cervical cancer screening *Significant at level 0.05 SR: Saudi Riyal; HPV: human papillomavirus

Factor	Undergoing cervical cancer screening		p-value
	Yes	No	
Age			< 0.001*
21-30 years	95 (12.7%)	651 (87.3%)	
31-45 years	178 (21.8%)	637 (78.2%)	
46-59 years	195 (31.1%)	432 (68.9%)	
60-65 years	49 (32.9%)	100 (67.1%)	
Education			< 0.001*
Intermediate school or less	20 (27.4%)	53 (72.6%)	
High school	82 (20.4%)	319 (79.6%)	
Bachelor’s degree or diploma	331 (20%)	1325 (80%)	
Higher education (Masters/PhD)	84 (40.6%)	123 (59.4%)	
Marital status			< 0.001*
Single	19 (4.7%)	382 (95.3%)	
Married	397 (23.7%)	1280 (76.3%)	
Divorced	68 (39.5%)	104 (60.5%)	
Widowed	33 (37.9%)	54 (62.1%)	
Working status			< 0.001*
Working	225 (22.6%)	769 (77.4%)	
Not working	204 (19.1%)	862 (80.9%)	
Retired	88 (31.8%)	189 (68.2%)	
Income			< 0.001*
Less than 5,000 SR	41 (11.7%)	310 (88.3%)	
5,000-10,000 SR	126 (17.8%)	580 (82.2%)	
10,001-15,000 SR	105 (19.3%)	440 (80.7%)	
15,001-20,000 SR	108 (30%)	252 (70%)	
Above 20,000 SR	137 (36.5%)	238 (63.5%)	
Place of residence			< 0.001*
Northern region	48 (25.3%)	142 (74.7%)	
Eastern region	139 (24.3%)	432 (75.7%)	
Western region	59 (13.7%)	373 (86.3%)	
Southern region	33 (11.6%)	251 (88.4%)	
Central region	238 (27.7%)	622 (72.3%	
Have you ever been diagnosed with any sexually transmitted disease?			< 0.001*
Yes	76 (66.7%)	38 (33.3%)	
No	441 (19.8%)	1782 (80.2%)	
Do you have any chronic disease (diabetes, hypertension, asthma, or any other chronic disease)?			< 0.001*
Yes	191 (34.8%)	358 (65.2%)	
No	326 (18.2%)	1462 (81.8%)	
Do you smoke?			< 0.001*
Yes	108 (40.3%)	160 (59.7%)	
No	409 (19.8%)	166 (80.2%)	
Have you had any gynecological problems before (abnormal vaginal bleeding, vaginal discharge, or other problems)?			< 0.001*
Yes	393 (31.6%)	852 (68.4%)	
No	124 (11.4%)	968 (88.6%)	
Did you receive the HPV vaccination?			< 0.001*
Yes	107 (60.5%)	70 (39.5%)	
No	410 (19%)	1750 (81%)	
Do you know someone who underwent cervical cancer screening?			< 0.001*
Yes	347 (53%)	308 (47%)	
No	170 (10.1%)	1512 (89.9%)	
Do you know someone who had cervical cancer?			< 0.001*
Yes	172 (42.5%)	233 (57.5%)	
No	345 (17.9%)	1587 (82.1%)	
Have you ever received a recommendation from a healthcare practitioner to undergo cervical cancer screening?			< 0.001*
Yes	381 (65%)	205 (35%)	
No	136 (7.8%)	1615 (92.2%)	
Knowledge levels			< 0.001*
Low knowledge level	382 (19.4%)	1583 (80.6%)	
Moderate knowledge level	98 (36.4%)	171 (63.6%)	
High knowledge level	37 (35.9%)	66 (64.1%)	

Age was significantly associated with undergoing cervical cancer screening (p <0.001), and it was observed that those aged between 21 and 30 years had a notably lower rate of undergoing cervical cancer screening compared to the other age groups. Education was significantly associated with undergoing cervical cancer screening (p <0.001), and it was observed that those with higher education (Masters/PhD) had a notably higher rate of undergoing cervical cancer screening compared to the other groups. Marital status was also significantly associated with undergoing cervical cancer screening (p <0.001), and it was observed that single participants had a notably lower rate of undergoing cervical cancer screening. Working status was significantly associated with undergoing cervical cancer screening (p <0.001), and it was observed that those who were not working had a notably lower rate of undergoing cervical cancer screening. Income was also significantly associated with undergoing cervical cancer screening (p <0.001), and it was observed that the higher the income, the higher the rate of undergoing cervical cancer screening. Place of residence was also significantly associated with undergoing cervical cancer screening (p <0.001), and it was observed that those from the southern region and those from the western region had a notably lower rate of undergoing cervical cancer screening. Being previously diagnosed with an STD was also significantly associated with a higher rate of undergoing cervical cancer screening (p <0.001) (66.7% vs. 19.8%). Having a chronic disease was also significantly associated with a higher rate of undergoing cervical cancer screening (p <0.001) (34.8% vs. 18.2%). Smoking was also significantly associated with a higher rate of undergoing cervical cancer screening (p <0.001) (40.3% vs. 19.8%). A history of gynecological problems was also significantly associated with a higher rate of undergoing cervical cancer screening (p <0.001) (31.6% vs. 11.4%). Receiving HPV vaccination, knowing someone who underwent cervical cancer screening, and receiving a recommendation from a healthcare practitioner to undergo cervical cancer screening were all positively and significantly associated with undergoing cervical cancer screening (p <0.001 in each association testing, respectively). Knowledge level was also significantly associated with undergoing cervical cancer screening (p <0.001), and it was observed that those with low knowledge levels had a notably lower rate of undergoing cervical cancer screening compared to the other groups.

Table 6 shows the multivariate logistic regression (factors predicting undergoing cervical cancer screening).

**Table 6:** Multivariate logistic regression (factors predicting undergoing cervical cancer screening) * Level of significance was set 0.05 SR: Saudi Riyal

Factor		p-value	Odds ratio	Confidence interval	
Age (21-30 years is the referent)					
31-45 years		0.229	1.28	0.86	1.92
46-59 years		0.029*	1.66	1.05	2.62
60-65 years		0.328	1.42	0.70	2.88
Education (intermediate school or less is the referent)					
High school		0.427	1.41	0.60	3.30
Bachelor’s degree or diploma		0.722	1.16	0.51	2.67
Higher education (Masters/PhD)		0.617	1.27	0.50	3.25
Marital Status (married is the referent)					
Divorced		0.054	1.63	0.99	2.68
Widowed		0.844	1.07	0.54	2.15
Working status (retired is the referent)					
Working		0.088	0.65	0.40	1.07
Not working		0.635	0.88	0.53	1.48
Income (less than 5,000 SR is the referent)					
5,000-10,000 SR		0.419	1.25	0.73	2.17
10,001-15,000 SR		0.367	1.31	0.73	2.33
15,001-20,000 SR		0.383	1.31	0.71	2.41
Above 20,000 SR		0.003*	2.58	1.39	4.79
Place of residency (northern region is the referent)					
Eastern region		0.428	1.24	0.73	2.14
Western region		0.971	0.99	0.54	1.80
Southern region		0.384	1.34	0.69	2.62
Central region		0.058	1.67	0.98	2.83
Previous diagnosis with sexually transmitted disease (yes vs. no)		0.191	1.49	0.82	2.72
Having chronic disease (yes vs. no)?		0.253	1.22	0.87	1.71
Do you smoke? (yes vs. no)		0.836	1.05	0.66	1.67
History of gynecological problems (yes vs. no)		< 0.001*	2.52	1.85	3.43
Knowing someone who underwent cervical cancer screening (yes vs. no)		< 0.001*	4.93	3.62	6.71
Knowing someone who had cervical cancer (yes vs. no)		0.193	0.78	0.54	1.13
Received a recommendation from a healthcare practitioner to undergo cervical cancer screening? (yes vs. no)		< 0.001*	13.00	9.58	17.64
Knowledge levels toward cervical cancer (low knowledge level is the referent)					
Moderate knowledge level		0.376	1.22	0.78	1.91
High knowledge level		0.962	0.98	0.50	1.94

The following factors were observed to be significantly predictive of undergoing cervical cancer screening: being 46 to 59 years of age (p = 0.013, odds ratio = 1.74, meaning a 74% increase in the rate of undergoing cervical cancer screening), having an income more than 20,000 SR (p = 0.003, odds ratio = 2.58, meaning a 158% increase in the rate of undergoing cervical cancer screening), having a history of gynecological problems (p <0.001, odds ratio = 2.52, meaning a 152% increase in the rate of undergoing cervical cancer screening), knowing someone who underwent cervical cancer screening (p <0.001, odds ratio = 4.93, meaning a 393% increase in the rate of undergoing cervical cancer screening), and receiving a recommendation from a healthcare practitioner to undergo cervical cancer screening (p <0.001, odds ratio = 13, meaning a 1300% increase in the rate of undergoing cervical cancer screening). Education, marital status, working status, place of residence, previous history of STD, having a chronic disease, smoking, knowing some who had cervical cancer, and knowledge level toward cervical cancer were all not significantly predictive of undergoing cervical cancer screening.

Table 7 displays the factors associated with knowledge about cervical cancer.

**Table 7:** Factors associated with knowledge about cervical cancer *Significant at level 0.05 SR: Saudi Riyal; HPV: human papillomavirus

Factor	Knowledge score		p-value
	Mean	Standard deviation	
Age			< 0.001*
21-30 years	3.79	3.37	
31-45 years	2.9	2.98	
46-59 years	2.36	2.59	
60-65 years	2.12	2.70	
Education			< 0.001*
Intermediate school or less	1.73	2.18	
High school	2.69	2.85	
Bachelor’s degree or diploma	2.98	3.05	
Higher education (Masters/PhD)	4.12	3.43	
Marital status			< 0.001*
Single	3.55	3.43	
Married	2.80	2.92	
Divorced	3.68	3.20	
Widowed	2.83	3.16	
Working status			< 0.001*
Working	3.34	3.27	
Not working	2.87	2.93	
Retired	2.23	2.56	
Income			< 0.001*
Less than 5,000 SR	2.43	2.76	
5,000-10,000 SR	2.51	2.56	
10,001-15,000 SR	3.03	3.19	
15,001-20,000 SR	3.76	3.42	
Above 20,000 SR	3.62	3.35	
Place of residency			< 0.001*
Northern region	2.49	2.04	
Eastern region	3.89	3.32	
Western region	2.36	2.85	
Southern region	2.52	2.93	
Central region	2.98	3.07	
Have you ever been diagnosed with any sexually transmitted disease?			< 0.001*
Yes	5.34	3.40	
No	2.87	2.99	
Do you have any chronic disease (diabetes, hypertension, asthma, or any other chronic disease)?			0.451
Yes	3.08	3.15	
No	2.97	3.03	
Do you smoke?			< 0.001*
Yes	4.51	3.53	
No	2.80	2.94	
Have you had any gynecological problems before (abnormal vaginal bleeding, vaginal discharge, or other problems)?			< 0.001*
Yes	3.21	3.12	
No	2.75	2.97	
Did you receive the HPV vaccination?			< 0.001*
Yes	5.36	3.44	
No	2.80	2.94	
Have you ever undergone cervical cancer screening (pap smear with or without HPV screening)?			< 0.001*
Yes	4.15	3.29	
No	2.66	2.91	
Do you know someone who had cervical cancer?			< 0.001*
Yes	4.18	3.28	
No	2.53	2.84	
Have you ever received a recommendation from a healthcare practitioner to undergo cervical cancer screening?			< 0.001*
Yes	5.09	3.38	
No	2.69	2.89	
If a doctor recommends it to you, would you undergo cervical cancer screening?			< 0.001*
Yes	3.10	3.08	
No	2.40	2.87	

Age was significantly associated with knowledge score toward cervical cancer (p <0.001), and it was observed that the younger the age group, the higher the knowledge score. Education was also significantly associated with knowledge score toward cervical cancer (p <0.001), where it was observed that the higher the education level, the higher the knowledge score. Marital status was also significantly associated with knowledge scores toward cervical cancer (p <0.001), and it was observed that married and widowed participants had notably lower knowledge scores. Working status was also significantly associated with knowledge score toward cervical cancer (p <0.001), and it was observed that those who were working had a notably higher knowledge score compared to the other groups. Income was also significantly associated with knowledge score toward cervical cancer (p <0.001), where it was observed that the higher the income level, the higher the knowledge score, with a similar knowledge score for those with an income higher than 15,000 SR. Place of residence was also significantly associated with knowledge score toward cervical cancer (p <0.001), where it was observed that those from the eastern region had a notably higher knowledge score compared to participants from other regions. Being previously diagnosed with a sexually-transmitted disease, smoking, having a history of gynecological problems, receiving HPV vaccination, undergoing cervical cancer screening, knowing someone who had cervical cancer screening, and receiving a recommendation from a healthcare practitioner to undergo cervical cancer screening all had a significantly positive association with undergoing cervical cancer screening (p <0.001 in each association testing, respectively). Moreover, willingness to undergo cervical cancer screening if it was recommended by a doctor was also significantly associated with a higher knowledge score (p <0.001).

## Discussion

The presence of an efficient screening test as well as the availability of an effective vaccine have turned cervical cancer into a preventable disease [31]. For the sake of utilizing these interventions and diminishing the mortality and morbidity caused by cervical cancer in Saudi Arabia, the current status of cervical cancer screening prevalence, its predictive factors, HPV uptake rate, and knowledge and attitude about cervical cancer in the nation must be evaluated. Identifying these issues will allow real-time, nationwide strategic planning and implementation of programs for cervical cancer. Conducting this research, as aforementioned, stemmed from the desire to aid in actualizing these benefits and getting closer to eliminating cervical cancer in Saudi Arabia. In order for this study to give a holistic and comprehensive view of the topic, participants from all over the nation with varying sociodemographic characteristics were included.

In this study, the prevalence of cervical cancer screening was found to be 22.1%. Nationally, this rate is higher than what was found in a study done in Al-Madinah in 2021 (12.5%) [31] and higher than what was found in Al-Qassim in 2019 [32] (15.3%). However, it was found to be similar to a study in Riyadh in 2018 (26%) [23] and lower than what was found in Jeddah in 2021 (33.4%) [22]. The rate of cervical cancer screening among Saudi women in this study (22.1%) was notably lower when compared to other Arab countries, Kuwait (35.2%) [33], and Jordan (31.2%) [34]. When compared to other countries, the cervical cancer screening rate in Saudi Arabia based on this study was found to be lower than the rates in Jamaica (40.7%) [35], China (63.7%) [36], Georgia (67%) [37], the United Kingdom (72%) [38], Brazil (73%) [37], Austria (80%) [37], and the United States (93%) [39]. However, the rate of cervical cancer screening among Saudi women in this study (22.1%) was higher when compared to Ethiopia (17.8%) [40], Nepal (15.7%) [41], Ghana (12%) [42], Uganda (4.8%) [43], India (3.9%) [44], and Cameroon (3.5%) [45]. Rates similar to those found in this study were found in Tanzania (21%) [46] and Turkey (21.9%) [47]. The reason behind varying rates of cervical cancer screening prevalence across different countries can be multifactorial. The strength of the healthcare system, the practice of healthcare practitioners and their adherence to recommended screening tests, the cost of the test, the availability of the test, the social familiarity of the test, and the level of awareness toward cervical cancer and its screening are all hypothesized to play a role in the prevalence of cervical cancer screening. In this study, 66% of those who underwent cervical cancer screening reported they did it because a healthcare provider recommended it; 42.4% reported they did not undergo the test because it was never recommended to them; 85% reported that they would do it if it was recommended to them; and 74.9% reported they never received a recommendation from a healthcare practitioner to undergo cervical cancer screening. This reflects a low rate of uptake despite a high level of acceptance. This discrepancy is most likely due to the clearly demonstrated weak application of the cervical cancer screening guidelines, most probably from the side of primary health care providers. Therefore, we highly encourage initiating programs that encourage and mandate primary healthcare physicians to recommend cervical cancer screening. In addition to that, we addressed the considerable rate of not undergoing the test due to lack of knowledge (14.8%), believing they are not at risk (14.5%), and finding the procedure awkward, potentially painful, or harmful, which indicates a decreased level of awareness regarding cervical cancer screening. Not to mention that when assessing knowledge about cervical cancer, (84.1%) had a low knowledge level. These findings shed light on the importance of conducting social educational campaigns that target women to raise awareness and eliminate faulty beliefs.

The level of HPV vaccine uptake in this study was found to be 7.6%, which, although considered low, is higher than what was previously reported by a study in Saudi Arabia in 2022 (0.02%) [31] and higher than what was found in another national study in Riyadh (1%) [23]. The rate of HPV vaccine in Saudi Arabia remains suboptimal and lower than what is observed in the United Kingdom (ranging from 44% to 93.4%) [48], Malaysia (77%) [49], the USA (ranging from 56.1% to 60.4%) [50], and Egypt (19.9%) [51]. The lower rate of HPV vaccine uptake when compared to the other counties is thought to be mainly because of the absence of national vaccination programs until recently and the lack of knowledge about cervical cancer and the HPV vaccine. However, we expect the rate of HPV vaccines to exponentially grow in the next decade, as the Saudi Ministry of Health recently initiated an elective immunization schedule for HPV vaccines for boys and girls aged between nine and 12 years. As vaccine uptake is elective and mainly dependent on the consent of legal guardians, the success of this initiative is questionable. This guarded view regarding the success of the initiative can be assured, as 79.3% of the participants reported that if their children were eligible for the vaccine and it was offered to them, they would let them take it. However, we again highly recommend intensifying the educational campaigns that target the general population and, in particular, young and middle-aged parents who are highly likely to have children aged between nine and 12 years to secure an uptake rate that is as high as possible.

In this study, although many factors were found to be significantly associated with undergoing cervical cancer screening, when adjusting for confounders using multivariate logistic regression, only the following were observed to be predictive factors: age 46-59 years (raised the screening rate by 74%), income above 20,000 SR (raised the screening rate by 158%), history of gynecological problems (raised the screening rate by 152%), knowing someone who underwent cervical cancer (raised the screening rate by 393%), and receiving a recommendation from a healthcare practitioner to undergo cervical cancer screening (raised the screening rate by 1300%). Age might have increased the rate of cervical cancer screening due to being more concerned about health as well as having more knowledge and experience with gynecological problems. High income might be associated with better health practices and access to better healthcare services received from health insurance rather than the governmental healthcare system. A history of gynecological problems will most probably lead to seeking help from either family physicians or gynecologists, who, when assessing the case, might offer the test and thus increase the rate of screening. Knowing some who underwent cervical cancer screening increases the familiarity of the test and thus its acceptance; it may as well positively influence females and make them seek the screening service. The strongest determinant of cervical cancer screening is having it recommended by a healthcare practitioner. This is probably because it is a safe, painless, and relatively quick procedure that has the potential to discover and prevent a deadly disease, and when the patients receive this information in an appropriate manner, they will accept it smoothly. The positive influence of some factors previously reported in the literature includes age [40, 52], income [45], and having the test recommended by a physician [22, 34]. In this study, a history of sexually transmitted disease was not predictive of undergoing cervical cancer screening; however, previous studies showed that testing for HIV is predictive of undergoing cervical cancer screening [40]. Surprisingly, smokers were observed to have undergone the screening significantly more than non-smokers; despite that, this association was not predictive. This could possibly be due to smokers trying to compensate for their negative health attitudes. Having a chronic disease was also significantly associated with higher rates of screening; however, this association was also not predictive. This could possibly be explained by participants being offered the screening during their routine follow-up as well as being more cautious about their health due to the comorbidities.

In this study, the mean knowledge score was 2.99 (only 23% of the total score), and as mentioned before, 84.1% had a low knowledge level (less than 50% of the total score). This is even lower than the findings of the study done in Al-Madinah and Al-Munawara in 2023, where the knowledge level was also low, but the median knowledge score was about 40% of the total score [31]. This study also had lower knowledge (measured in mean knowledge percentage out of 100%) when compared to Cameroon (26.2%) [53], Oman (38.3%) [54], Kuwait (54%) [55], and the United Arab Emirates (66.2%) [56].

In this study, many factors were observed to be significantly associated with knowledge regarding cervical cancer; however, the highest knowledge scores were observed in participants who previously were diagnosed with sexually transmitted diseases (score = 5.34), those who received HPV vaccine (score = 5.36), and those receiving a recommendation from a healthcare practitioner to undergo cervical cancer screening (score = 5.09) (note that the maximum score is 13). This sums up that the greatest influence on knowledge toward cervical cancer is previous encounters and consultations related to cervical cancer screening, history of sexually transmitted disease, and receiving the HPV vaccine, emphasizing the great role of physicians in healthcare centers to raise awareness and advocate health-promoting behavior.

Strengths and limitations

The design of this study (cross-sectional) and the mean of data collection (self-administered) bear innate downsides, such as the possibility of recall bias; this is possibly the only limitation of this study. As for its strengths, this study's sample size is considered to be high, as it was more than what was required to achieve a high confidence level of 99.9% and a low margin of error of 4%. In addition to that, despite the high variability of Saudi Arabian female characteristics (in terms of age, marital status, income level, education level, and place of residence), a sufficient number from each group of these characteristics participated in the study, making its results highly reliable and representative of the true status in the country and qualifying it to be labeled as a nationwide study. Moreover, this study navigated the topic of cervical cancer screening and the issues related to it in depth and yielded a comprehensive layout. In short, this study provides a solid and reliable baseline for the current prevalence of cervical cancer screening and its predictive factors. In addition, the study covered related issues such as the HPV vaccine uptake rate and knowledge and attitude toward cervical cancer, its screening, and the HPV vaccine.

Recommendations

Due to the abovementioned demonstration of the poor current status and underlying cause, we highly recommend that the health authorities in the nation start initiatives that encourage healthcare practitioners to educate the target population about cervical cancer, offer cervical cancer screening, and provide HPV vaccines for eligible patients. In addition to that, we highly recommend conducting educational campaigns in schools, universities, workplaces, and in public to educate the target population about cervical cancer and its prevention and promote health-seeking behavior.

## Conclusions

This study reflects low levels of cervical cancer screening rates (22.1%) and HPV vaccine uptake (7.6%). There is also a low level of knowledge about cervical cancer screening (84.1%), despite the positive attitudes reflected in the high acceptance rate towards cervical cancer screening if it was provided by a physician (85%), a high acceptance rate toward HPV vaccine if it was offered and they were eligible (80.5%), and a high acceptance rate for giving the participants’ children the HPV vaccine if it was offered and they were eligible (79.3%). These findings clearly show the gaps that led to poor practices toward cervical cancer, which are inadequate communication between the physicians and the target population, the shortage of enough educational campaigns toward the target population, the lack of programs and initiatives that encourage the target population to take action, and the deficiency in healthcare providers' practice toward cervical cancer screening.
